# Leaf color variation mechanism of the yellow-to-green mutant ‘*ytg-2*’ in *Phaseolus vulgaris* L.

**DOI:** 10.1270/jsbbs.24018

**Published:** 2025-06-11

**Authors:** Ziyan Wang, Dajun Liu, Xiaoxu Yang, Ruijie Ji, Guojun Feng, Zhishan Yan, Chang Liu

**Affiliations:** 1 Academy of Morden Agriculture and Environmental Sciences, Heilongjiang University, Harbin 150000, China; 2 Jiaxiang Industrial Technology Research Institute of Heilongjiang University, Jining, 272400, China

**Keywords:** snap bean (*Phaseolus vulgaris* L.), leaf color mutant, yellow-to-green, transcriptome, chlorophyll

## Abstract

Snap bean leaves are the primary site of photosynthesis. Mutant leaf color provides valuable tools for investigating leaf color changes, their role in photosynthesis, and pigment metabolic pathways. In this study, we found that the seedling leaves of ‘*ytg-2*’ exhibited a yellow phenotype characterized by reduced chlorophyll content compared with ‘A18’. Blockage of the uroporphyrinogen III (Urogen III) to the fecal porphyrinogen III (Coprogen III) synthesis pathway primarily affected the synthesis of chlorophyll intermediate metabolites. As the plants matured, their leaves transformed from yellow to light green, accompanied by an increase in the total chlorophyll content. Transcriptome analysis revealed that the significantly down-regulated expression of four genes in the HemE gene family (*Phvul.008G059400.1*, *Phvul.010G110900.1*, *Phvul.008G147200.1*, and *Phvul.010G069500.1*), which blocked the Urogen III to Coprogen III conversion, was the primary cause of the yellow phenotype in ‘*ytg-2*’ seedlings. Additionally, the down-regulation of *POR* (*Phvul.004G113000.1*) and *NOL* (*Phvul.004G163900*) genes during the chlorophyll cycle explained the reduced total chlorophyll content in ‘*ytg-2*’ and the gradual normalization of the chlorophyll a/b ratio in ‘*ytg-2*’ yellow leaves. The expression of *PAO* (*Phvul.004G163900.1*), a key enzyme in chlorophyll degradation, further confirmed that the yellow-to-green transition in ‘*ytg-2*’ leaves was linked to chlorophyll degradation processes.

## Introduction

Snap bean, a globally important legume crop belonging to the *Phaseolus* genus, depends heavily on photosynthesis for biomass accumulation and economic yield. Leaves, as the primary sites of photosynthesis, play a critical role in this process. Although leaf color mutants are often viewed as detrimental owing to their direct or indirect impact on photosynthesis and subsequent yield reduction, advancements in molecular biology and functional genomics have unlocked their potential for research. These mutants serve as valuable biomaterials for investigating photosynthetic mechanisms and gene functions, offering a theoretical foundation for enhancing the yield potential of snap beans.

Leaf color mutants exhibit wide prevalence among plant species and are important for elucidating various aspects of chlorophyll biosynthesis, chloroplast development, nucleoplasm gene interactions, gene function identification, and signal transduction. Researchers at national and international levels have acquired such mutants from a variety of crops including wheat (Falbel and Staehelin 1994), rice, Arabidopsis (Waters *et al.* 2009), Rape (Chu *et al.* 2015), and soybean (Campbell *et al.* 2015). These mutants have been extensively investigated to gain insights into pigment content metabolism, photosynthetic physiology, chloroplast development, and gene cloning (Ma *et al.* 2017, Zhu *et al.* 2016). These studies have contributed fundamental knowledge essential for revealing the mechanisms of leaf color mutation, chlorophyll synthesis pathways, and the excavation of related genes. Snap bean (*Phaseolus vulgaris* L.), a crucial crop cultivated globally within the Leguminosae family, plays a pivotal role in bioaccumulation and economic yield, primarily driven by photosynthesis. Considering that leaves serve as the main site for photosynthesis, any direct or indirect impact of leaf color mutations on this process can result in reduced crop yields, commonly referred to as unfavorable mutations. Advancements in biotechnology, such as molecular biology and functional genomics, have facilitated the extensive utilization of leaf color mutants in theoretical and fundamental research. The application of snap bean leaf color mutants as biological materials for the exploration of the photosynthesis mechanism and gene function identification of the crop holds the potential to establish a theoretical foundation for enhancing the yield potential of snap beans.

The biosynthesis of chlorophyll in higher plants is catalyzed by 15 enzymes, which are encoded by more than 20 genes. This intricate process commences from glutamyl-tRNA and terminates in the production of chlorophylls a and b (Tanaka and Tanaka 2006, Tanaka *et al.* 2011). Glutamate tRNA arises from the combination of glutamate and tRNA through biosynthesis, ultimately leading to the production of protoporphyrin IX (Proto IX) (Supplemental Table 1), the universal precursor of all tetrapyrroles and an intermediate metabolite in tetrapyrrole compound synthesis. Magnesium chelatase mediates the insertion of Mg^2+^ into Proto IX to yield magnesium protoporphyrin IX (Mg-Proto IX). It is crucial to note that any disruption in the enzymatic reaction halts the synthesis of chlorophyll a and b, ultimately resulting in leaf color mutations. Magnesium chelatase consists of three subunits, including magnesium chelatase H subunit (CHLH), magnesium chelatase I subunit (CHLI), and magnesium chelatase D subunit (CHLD), and exhibits functional activity only when all three subunits are concurrently present (Gibson *et al.* 1995, Papenbrock *et al.* 1997). Leaf color mutants resulting from mutations in genes encoding magnesium chelatase subunits have been documented in various crop species, such as Arabidopsis (Kobayashi *et al.* 2008, Rissler *et al.* 2002), rice (Jung *et al.* 2003, Zhang *et al.* 2006), wheat (Falbel and Staehelin 1996), and soybean (Campbell *et al.* 2015). (Deng *et al.* 2014) identified a spontaneous yellow-green leaf mutant, designated as ‘ygl7’, in the light-temperature-sensitive male sterile line PTGMS of rice. This mutation is controlled by a pair of recessive genes that encode CHLD. Notably, the ‘ygl7’ mutant demonstrated a relative increase in photosynthetic efficiency and no change in yield traits, despite of the reduction in chlorophyll content. Similarly, Li *et al.* (2016b) discovered a yellow-green leaf mutant named ‘siygl1’ in cereals, also encoding CHLD, akin to the rice ‘ygl7’ mutant. This mutant exhibited enhanced utilization of light energy compared with the wild type. These findings suggest that the mutated CHLD protein may not have completely lost its activity but instead acquired a new function. Both HemE and chlorophyll undergo a common synthesis process, commencing with glutamate and leading to protoporphyrin. Proto IX serves as a branching point for chlorophyll and HemE synthesis. Studies on tomato aurea (au) and yellow-green-2 (yg-2) mutants, which affect the photosensitized pigment chromophore synthesis pathway, have revealed that the inhibition of the HemE-photosensitized pigment chromophore pathway results in the excessive accumulation of HemE. This accumulation, in turn, inhibits the synthesis of alanine (ALA) in the chlorophyll synthesis pathway, thus triggering leaf color mutations (Terry and Kendrick 1996, 1999). The discovery of the “gun” series of nuclear-plastid genomic uncoupling mutants in *Arabidopsis thaliana*, the ‘gun1–6’ mutants, has provided crucial fundamental evidence supporting the role of Mg-proto IX as a signaling molecule involved in plastid signaling. GUN2, GUN3, and GUN5 encode HemE oxygenase (HO), photosensitive pigment chromophore synthase (phytochromobilin synthase), and CHLH, respectively, all contributing to tetrapyrrole synthesis (Mochizuki *et al.* 2008). Deletion of GUN2 and GUN3 leads to excessive HemE accumulation and inhibition of glutamyl-tRNA reductase activity, thereby impeding the synthesis of the chlorophyll synthesis precursor ALA. Consequently, the Mg-proto IX content decreased, while the expression of the nuclear-encoded photosynthetic gene (Lhcb) remained unaffected (Strand *et al.* 2003). GUN1 operates independently of the GUN2–6 pathway, which compensates for the loss of nuclear gene expression following treatment with the inhibitor lincomycin (LIN). GUN1 potentially plays a role in the plastid translation pathway at the early stage of seedling development (McCormac and Terry 2004).

Wu *et al.* (2007) reported existence of a rice mutant, denoted as ‘1ygl1’, with chlorophyll deficiency caused by a recessive mutation in the nuclear gene encoding chlorophyll synthase (CHLG). The mutant presented a yellow-green leaf phenotype during the early stages of plant development, which was characterized by reduced chlorophyll synthesis. Subsequently, the leaves turned yellow in the early reproductive stages and returned to a yellow-green hue in later stages. Remarkably, by the time of tasseling, the leaf color of the mutant reverted to a state closely resembling that of the wild type. Gao *et al.* (2021) reported a light green mutant, named Sumian 22 (cotton), which presented an A-T SNP mutation at the 317 bp position of the start codon of ABCI1 (ABC transporter protein I family member 1). In the young stage, the leaves of Sumian 22 appeared yellow and gradually changed to a light green color during the boll stage. At the maturity stage, the mutant leaves turned green but still showed slight differences compared to the wild type. Mao *et al.* (2019) reported a light green light-sensitive mutant in cotton, referred to as ‘vsp’, and attributed its occurrence to a 201-bp base sequence deletion in the second exon of the GhPUR4 gene. This deletion impaired the normal function of formyl glycine rib peptide aminotransferase (FGAMS) in the fourth step of the ab initio purine biosynthetic pathway, resulting in abnormal development of ‘vsp’ chloroplast thylakoid, as well as a reduction in photosynthetic capacity due to impaired chlorophyll synthesis. ‘vsp’ mutants exhibited light green young leaves under normal field conditions, with a more pronounced phenomenon of a 2-d leaf development period. Over time, the leaves gradually transitioned from yellow to green when reaching maturity. PDS, ZDS, CRTISO, and β-OsLCY are important enzymes in the ABA and carotenoid biosynthetic pathways. Their activities are regulated by ABA and carotenoid synthesis (Lu *et al.* 2020). Leaf whitening in mutant ‘phs’ was due to a reduction in the amount of ABA, photoinhibition in the photosystem, and significant down-regulation of the PSII core proteins CP43, CP47, and D1 (Fang *et al.* 2008).

Snap bean is a globally cultivated leguminous vegetable crop with tender pods as edible organs. Among the varieties, ‘A18’ is a dwarf-type vegetable bean with green stems and leaves, as well as yellow pods. It is particularly popular in northern China and has been widely cultivated. Snap bean leaves are primary sites for photosynthesis, supplying essential nutrients for plant growth and development. Changes in leaf color can significantly affect snap beans throughout their growth cycle. However, the specific genes regulating chlorophyll synthesis that drive the transition from yellow to green during the reproductive period remain unclear. Therefore, in this study, we used ‘A18’ seeds as the material for ^60^Co-γ radiation mutagenesis to generate a mutant with yellow-to-green leaves, named ‘*ytg-2*’. To reveal the physiological and molecular mechanisms of the yellow-to-green mutant, we performed physiological analyses and transcriptome studies on both mutant and wild-type plants to screen for candidate genes that affect the yellow-to-green leaf phenotype. These findings provide valuable insights into the mechanisms of chlorophyll biosynthesis in this yellow-to-green mutant and offer a foundation for future research on breeding, variety improvement, and gene localization.

## Materials and Methods

### Test materials

The snap bean cultivar ‘A18’ is a self-incompatible line with high generational stability in inheritance. Using ‘A18’ as the wild type, its dried seeds were exposed to ^60^Co-γ radiation at 150 Gy to obtain the stably inherited yellow-to-green leaf mutant, named ‘ytg-2’. A stable yellow-to-green mutant plant was obtained after several generations of self-pollination, with its primary leaves initially appearing yellow before gradually turning green. Both the wild-type ‘A18’ and the yellow-to-green mutant ‘*ytg-2*’ were cultivated in a cold shed at Heilongjiang University, Heilongjiang Province, China, throughout the year of 2021.

### Design of field experiments

The first trifoliate leaves of both species were tagged after emergence of the first trifoliate leaf. Subsequently, the first trifoliate leaves at 0, 7, 14, 21, 28, 35, and 42 d after tagging were selected for sampling, respectively. The samples were snap-frozen in liquid nitrogen and stored at –80°C for the determination of photosynthetic pigment content, photosynthetic parameters, and chlorophyll precursors. Furthermore, the chlorophyll content, chlorophyll intermediate metabolites, and sequencing of the trans-greenome were measured on the leaves at the 7, 28, and 42 d after the onset of yellowing, trans-greening, and late trans-greening, respectively. Each measurement was performed in triplicate.

#### Determination of agronomic traits

After the mutant ‘*ytg-2*’ and the wild-type ‘A18’ grew the first trifoliate leaves, 10 plants of each material were selected for agronomic trait investigation. Nutritional growth indices of the plants, including plant height, main stem thickness, leaf length, leaf width, single fresh weight, and single dry weight, were measured every 7 d. The measurements were performed in triplicate, and the average value was taken after the measurements. The cotyledon nodes of bean plants, along with the aforementioned parts, were measured using a tape measure. Stem thickness at the cotyledon node was measured using a vernier caliper. The length and width of the first trifoliate leaf were recorded using a tape measure, and the area was calculated using the coefficient method. The fresh weight of the plants above the cotyledon nodes was determined using a 1/100 electronic balance. Plants were blanched in an oven at 110°C for 20 min and then dried at 80°C until their weights stabilized, after which the dry weight of each plant was measured. At the stage of full maturity, three healthy mutant ‘*ytg-2*’ plants and three wild-type ‘A18’ plants were selected. Parameters such as single plant yield, pod length, pod width, number of pods per plant, and 100-grain weight were measured and recorded. The yield of fresh pods was determined using a 1/100 electronic balance, and the number of seeds per 100 pods was counted. Additionally, pod length and width were measured using a vernier caliper.

#### Determination of photosynthetic pigment content and photosynthetic parameters

Photosynthetic pigment content was determined following the method described by Arnon (1949). Photosynthetic parameters were measured using a hand-held photosynthesizer on days 7, 14, 21, 28, 35, and 42. The measured indices included net photosynthetic rate (Pn), intercellular CO_2_ concentration (Ci), transpiration rate (E), and stomatal conductance (Gs).

#### Determination of chlorophyll intermediate metabolites

Chlorophyll intermediate metabolite content was quantified in the first trifoliate leaves of both the mutant ‘*ytg-2*’ and the wild-type ‘A18’ plants at 7, 28, and 42 d. A 0.5 g sample of the first trifoliate leaf, consisting of three leaflets, was collected, rapidly frozen in liquid nitrogen, and stored at –80°C for further analysis. The levels of the chlorophyll intermediate metabolites were measured using a spectrophotometer. ALA, PBG, Urogen III, and Coprogen III were quantified according to the methods described by Bogorad (1962) and Beaven (1975). Proto IX, Mg-Proto IX, and protochlorophyllate (Pchlide) levels were determined using the methods described by Rebeiz *et al.* (1975) and Masuda *et al.* (2003).

### Transcriptome sequencing analysis

Leaves from both ‘A18’ and mutant ‘*ytg-2*’ at the yellowing, greening, and late greening stages of 7, 28, and 42 d were used as experimental materials. The leaves were wrapped in tinfoil and stored in a freezer at –80°C. Following RNA extraction from the samples, libraries were generated and sequenced by Lianchuan Biotechnology (Hangzhou, China). After sequencing, Lianchuan Biotechnology applied Cutadapt to filter out ineligible sequences, which ensured the acquisition of valid data. Unwanted raw reads were eliminated, including reads with a proportion of unidentifiable base information greater than 5% and low-quality reads (defined as reads where the number of bases with quality values (Q) below 10 constituted more than 20% of the total reads). Subsequently, a reference genome comparison was performed on the pre-processed valid data using Hisat2.

Based on the comparison results of Hisat2, FPKM was used as a measure of gene expression. DEGs were identified based on the following criteria: fold-change (FC) ≥2. FC ≤0.5 was the threshold of change and its q-value was <0.05. DEGs were then analyzed for Gene Ontology (GO) and Kyoto Encyclopedia of Genes and Genomes (KEGG) enrichment analyses. GO enrichment analysis first compared all DEGs against the GO gene pool and then determined the number of GO-enriched genes in each category. Hypergeometric tests were performed to evaluate the significance of GO terms, with corrected P-values (P_adjust_) <0.001 compared to the whole genomic background. This analysis revealed the key biological processes, cellular components, and molecular functions associated with DEGs. Moreover, KEGG, a database focused on metabolic pathways, was used. By applying hypergeometric tests, significantly enriched pathways were identified among significant DEGs compared to the whole genomic background using a corrected P-value ≤0.05. KEGG enrichment analysis identified the main metabolic and signaling pathways involved in DEGs.

### Validation of transcriptome sequencing results through qRT-PCR

RNA extraction (Supplemental Text 1): Total RNA was extracted using Total Plant Trizol Reagent (Thermo Fisher, 15596018) following the manufacturer’s protocol. The quality and integrity of RNA samples were assessed using an Agilent 2100 Bioanalyzer and 1% agarose gel electrophoresis.

qRT-PCR Analysis: qRT-PCR was conducted using the SYBR Premix Ex Taq^TM^ kit (Takara Biotechnology, Dalian, China) in a 20-μL reaction system, following the manufacturer’s instructions. The three-step reaction procedure was as follows: 95°C for 5 min, followed by 40 cycles of 95°C for 10 s, and 58°C for 1 min. Primers for qRT-PCR were designed using Primer 5.0, with the actin gene selected as the internal reference (Table 1). Each reaction was performed in triplicate. The gene expression levels were calculated using the 2^–ΔΔCT^ method.

cDNA synthesis: The first strand of cDNA was synthesized using an RNA Reverse Transcription Kit (Tiangen, China) and stored at –20°C for future use.

## Results

### Identification of mutant *ytg-2* phenotype and agronomic traits

**Fig. 1.**
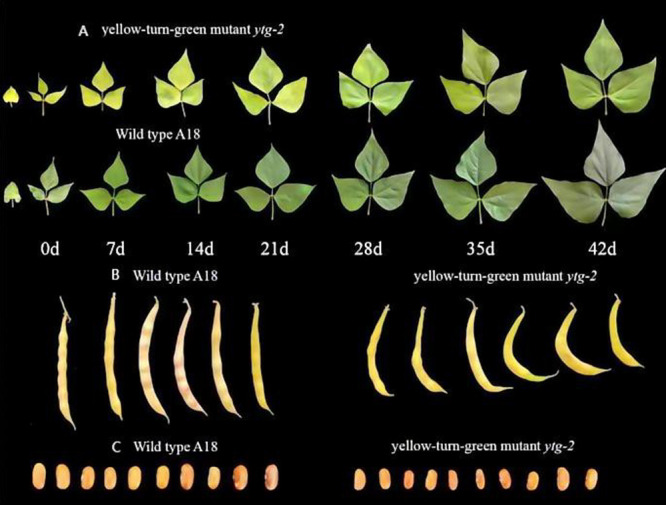
Field phenotype survey of wild-type A18 and yellow-turn-green leaf mutant ‘*ytg-2*’. A) Leaf phenotype comparison between wild-type A18 and yellow-green leaf mutant *ytg-2*; B) Pod phenotype comparison between wild-type ‘A18’ and yellow-turn-green leaf mutant ‘*ytg-2*’; C) Seed phenotypes of wild-type and yellow-turn-green leaf mutant at different developmental stages. From left to right are the leaf phenotypes at 0, 7, 14, 21, 28, 35, and 42 d of the first true leaf and the first three compound leaves.

Field phenotype survey suggested that the mutant ‘*ytg-2*’ demonstrated a yellowing phenotype upon the emergence of the first primary true leaves. However, as the reproductive process progressed, a noticeable transformation occurred, in which the lower leaves gradually transitioned from yellow to green. The duration of this yellow-to-green leaf cycle was approximately 42 d. From the first trifoliate leaf emergence to the complete greening of the leaf, it can be divided into three stages: yellowing (0–21 d), greening (21–35 d), and late greening (35–42 d) (Fig. 1C). The mutant ‘*ytg-2*’ displayed inferior performance than the wild-type ‘A18’ in the early stage of growth, as evidenced by significantly reduced agronomic traits. However, as the leaves transitioned from yellow to green, their growth potential gradually began to be restored. All agronomic trait indicators exhibited significant reductions, manifesting in curled pods (Fig. 1B). The seeds were not full and crumpled (Fig. 1).

**Fig. 2.**
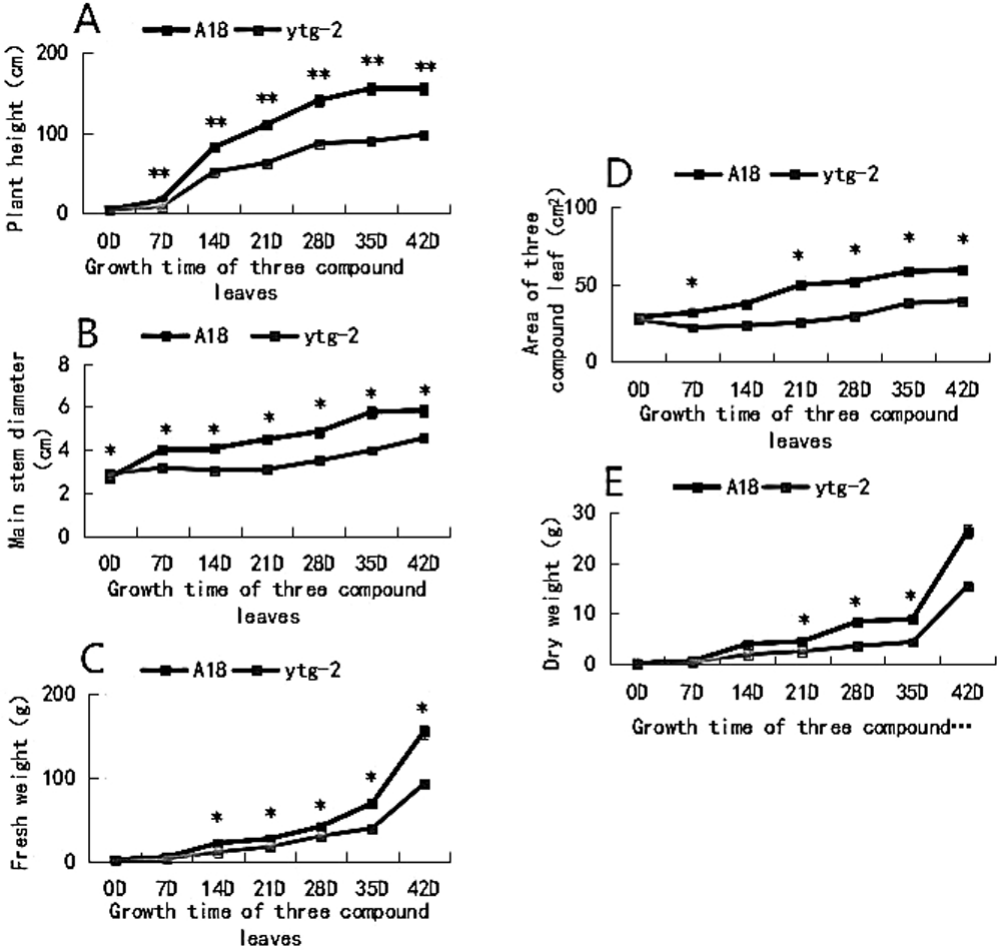
Growth curves of the yellow-turn-green leaf mutant ‘*ytg-2*’ and its wild-type ‘A18’ in different developmental stages of snap bean. A) plant height; B) main stem diameter; C) fresh weight; D) the dry weight; E) leaf area. Note: *: P < 0.05, **: P < 0.01.

The analysis of Fig. 2 revealed noteworthy observations regarding the mutant ‘*ytg-2*’. Initially, prior to the commencement of the observation, no significant differences were observed in various indicators (plant height, main stem thickness, trifoliate leaf area, fresh weight, and dry weight) compared to the wild-type ‘A18’ wild-type. However, the mutant ‘*ytg-2*’ demonstrated a slower growth rate and larger differences in the indicators compared to the wild-type ‘A18’ at the yellow leaf stage. Following the greening stage, plant growth potential gradually recovered. At the late greening stage, all measured indexes remained significantly lower than those of the wild-type ‘A18’. The final plant height, main stem thickness, trifoliate leaf area, fresh weight, and dry weight were 62.88%, 77.74%, 66.18%, 59.46%, and 58.77%, respectively, of those of the wild-type ‘A18’, which demonstrated substantial divergence.

Furthermore, various yield-related traits such as pod length, pod width, yield per plant, number of pods per plant, number of seeds per pod and 100-grain weight of the wild-type ‘A18’ were all greater than those of the mutant ‘*ytg-2*’ (Table 2). Compared with the mutant ‘*ytg-2*’, the pod length and pod width of the wild-type ‘A18’ increased by 28.91% and 6.43%, respectively; the yield per plant, number of pods per plant, number of seeds per pod, and 100-grain weight increased by 45.85%, 50.76%, 63.64% and 39.33%, respectively, with highly significant differences except for pod width. In summary, leaf color variation affected both the growth and development of mutant plants, leading to restricted nutritional growth. Compared to the wild-type ‘A18’, the reproductive growth of the mutant ‘*ytg-2*’ was also affected, resulting in poor performance in yield-related traits.

### Analysis of photosynthetic pigments, chlorophyll intermediate metabolites content, and photosynthetic parameters

**Fig. 3.**
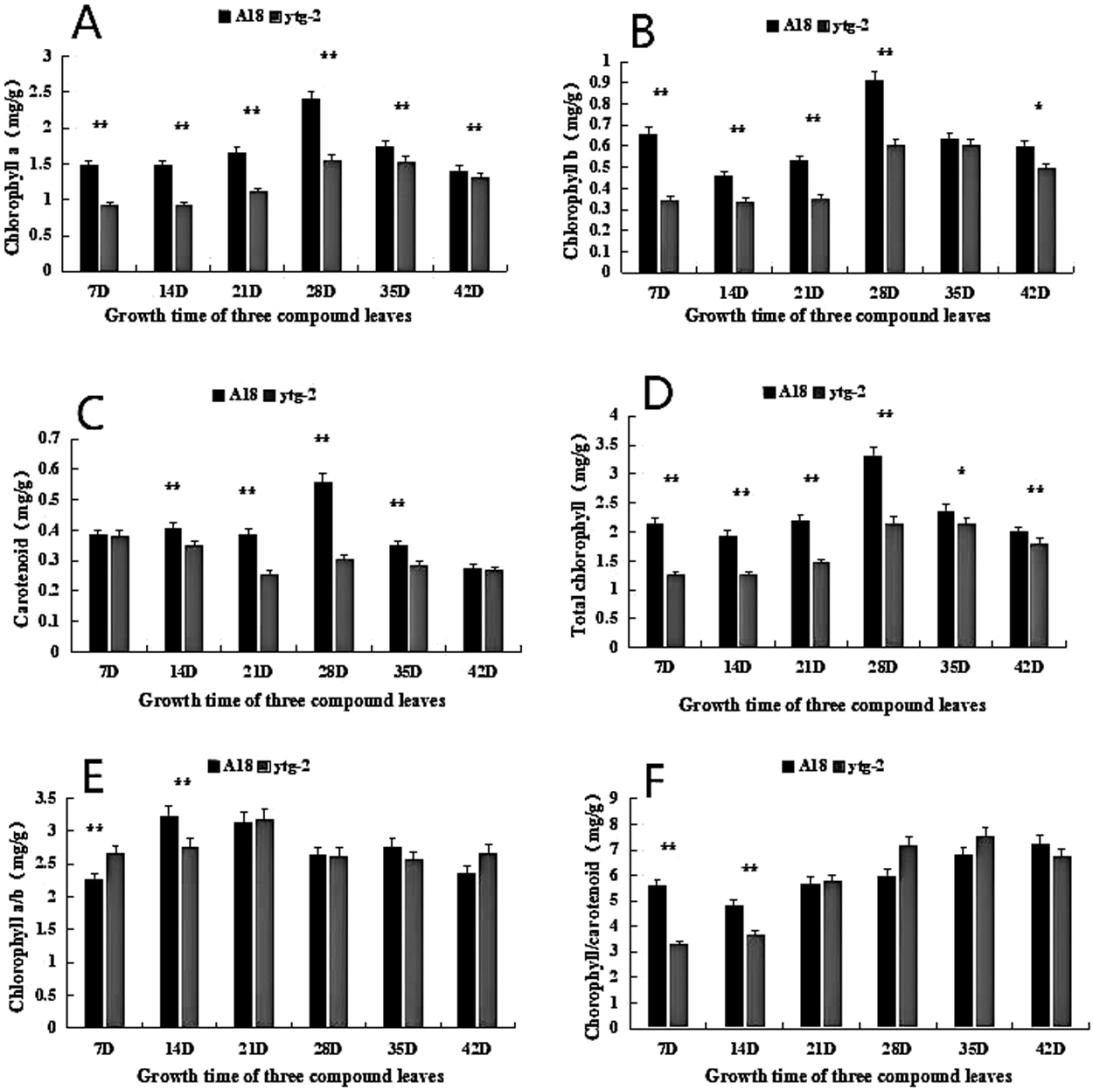
The photosynthetic pigment content between the yellow-turn-green leaf mutant ‘*ytg-2*’ and its wild-type ‘A18’ at different developmental stages. A) chlorophyll a content; B) chlorophyll b content; C) carotenoid content; D) Total chlorophyll content; E) the chlorophyll a/b value; F) chlorophyll/carotenoid value. Note: *: P < 0.05, **: P < 0.01.

As shown in Fig. 3, during the observation period (0–42 d), the mutant ‘*ytg-2*’ exhibited a significant decrease in total chlorophyll and carotenoid content compared to the wide-type ‘A18’. However, the magnitude of change was consistent with that observed in ‘A18’. This indicated that the main cause of leaf yellowing in mutant ‘*ytg-2*’ was its significantly reduced total chlorophyll content. To further investigate the cause of the yellow-to-green coloration of mutant ‘*ytg-2*’ leaves, we measured the relative contents of chlorophyll precursors ALA, PBG, Urogen III, Coprogen III, Proto IX, Mg-Proto IX, and Pchlide.

**Fig. 4.**
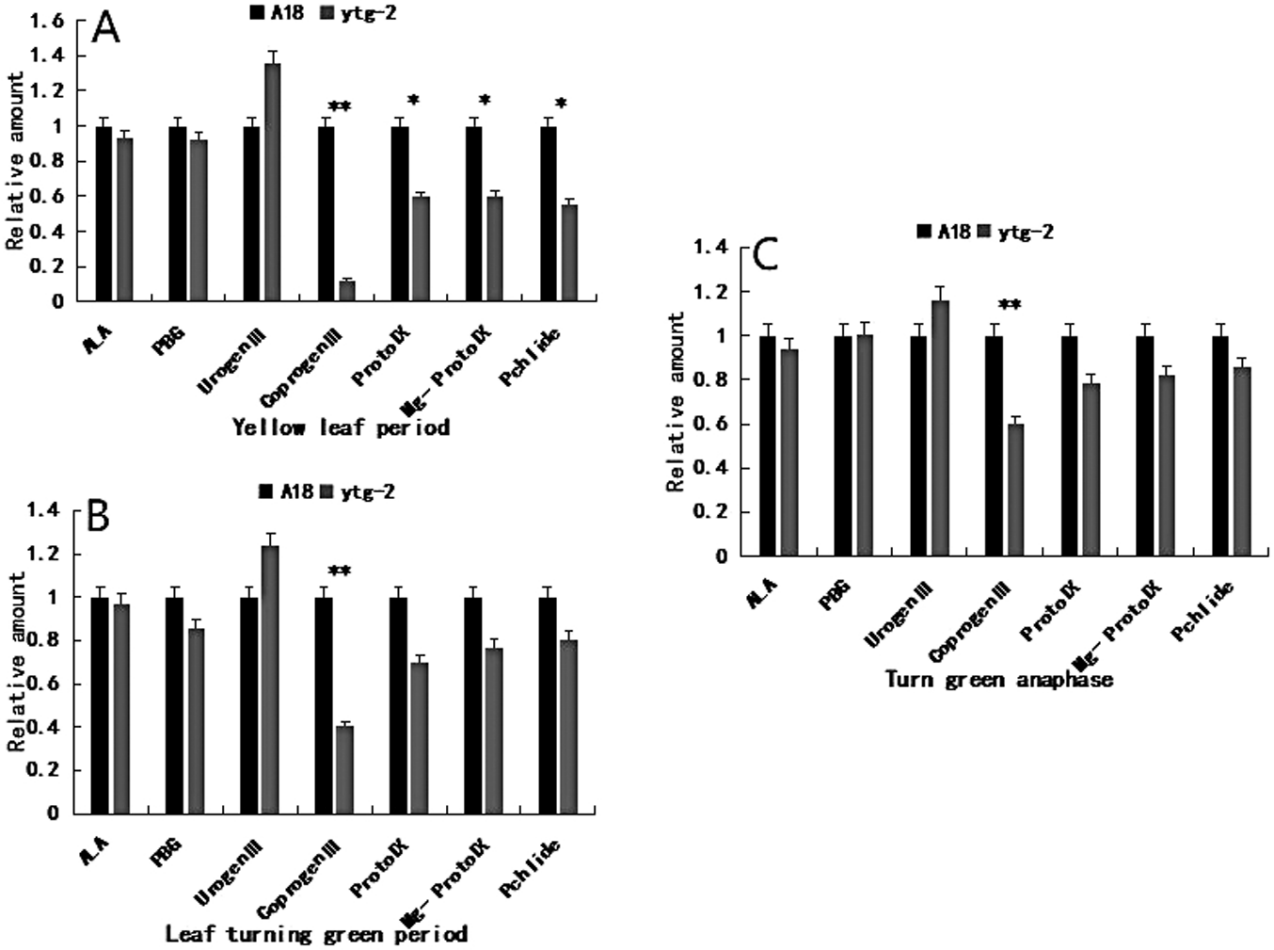
Comparison of intermediate chlorophyll metabolite content between the yellow-turn-green leaf mutant ‘*ytg-2*’and its wild-type ‘A18’ in the yellowing (A), green (B), and late green (C) stages. Note: *: P < 0.05, **: P < 0.01.

As shown in Fig. 4, the relative content of the precursor substance Urogen III of mutant ‘*ytg-2*’ was generally higher than that of the wide-type ‘A18’ and gradually decreased with the change in leaf color from yellow to green. The relative content of the precursor substance Coprogen III of mutant ‘*ytg-2*’ was significantly lower than that of the wide-type ‘A18’ throughout all three periods, with the difference in content gradually decreasing as the change in leaf color from yellow to green occurred. Moreover, the relative content of Proto IX, Mg-Proto IX, and Pchlide in the mutant ‘*ytg-2*’, following Coprogen III, was lower than that in the wide-type ‘A18’. However, these levels gradually increased with changes in leaf color from yellow to green. A significant difference was observed during the yellowing stage. This suggested that chlorophyll biosynthesis in mutant ‘*ytg-2*’ may encounter inhibition during the synthesis of Urogen III to Coprogen III. The blocked chlorophyll biosynthesis metabolism resulted in the reduction of chlorophyll in mutant ‘*ytg-2*’ leaves. However, the chlorophyll content increased in the later stages as the leaf yellow-to-green fertility process progressed.

**Fig. 5.**
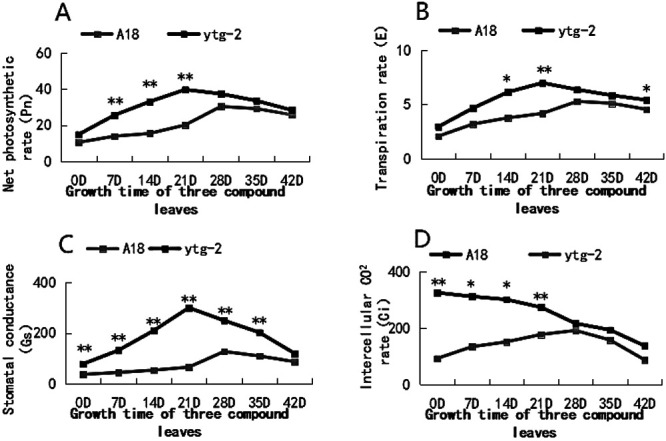
Comparison of photosynthesis parameters between the yellow-turn-green leaf mutant ‘*ytg-2*’ and its wild-type ‘A18’ at different developmental stages. A) net photosynthetic rate; B) stomatal conductance; C) transpiration rate; D) Intercellular CO_2_ concentration. Note: *: P < 0.05, **: P < 0.01.

As shown in Fig. 5, the mutant ‘*ytg-2*’ consistently exhibited lower values of Pn, Gs, and E than the wide-type ‘A18’, while Ci was always higher than the wide-type ‘A18’ during the observation period (0–42 d). Among them, Pn, Gs, and E of both the wide-type ‘A18’ and mutant ‘*ytg-2*’ demonstrated a single-peaked curve trend during the observation period of 42 d. They initially increased and then decreased with increasing growth time of the three-out compound leaves. The difference between them gradually increased until the 21st day and gradually decreased after the 21st day. Notably, Ci of the wide-type ‘A18’ showed an increasing and then decreasing trend, while the mutant ‘*ytg-2*’ indicated a decreasing trend and a negative correlation with its Gs until 28 d (mid-greening). The results demonstrated that the mutant plants possessed lower photosynthetic efficiency, and their stomata were closer to a greater extent than the wild-type plants. This suggests that under similar light conditions, the mutant plants may experience greater inhibition of photooxidation than the wild-type. The stomata of the plants were closed to act as a photoprotective agent.

### Illumina sequencing

In this study, to further elucidate the regulatory mechanism of leaf color change in the mutant ‘*ytg-2*’, we conducted Illumina sequencing, which yielded 826,184,300 valid reads. To ensure the reliability of the sequencing data, the distribution of different base contents was investigated in 18 samples (Table 3). The results indicated that the base content of GC ranged from 43.5% to 44.5%, the Q20 content was approximately 99.91%, and the variation trend was stable. These results demonstrate the high-quality and consistent nature of sequencing data, meeting the stringent requirements for reliable sequencing outcomes.

### Differentially expressed gene (DEG) analysis, functional annotation, and classification

All the obtained clean reads were compared to the reference genome. To quantify transcript expression levels, we applied normalization techniques that incorporated both mapped reads and transcript lengths. We adopted FPKM (Fragments Per Kilobase per Million) as a standardized measure of transcript or gene expression. In this study, we focused on three comparison groups: yellow leaf stage, yellow to green leaf stage, and green leaf stage (YL *vs.* YLCK, YGL *vs.* YGLCK, GL *vs.* GLCK), resulting in the identification of 7761, 6186, and 5847 DEGs, respectively. Among them, the YL *vs.* YLCK comparison group exhibited 3438 up-regulated and 4391 down-regulated DEGs, whereas the YGL *vs.* YGLCK comparison group revealed 2564 up-regulated and 3734 down-regulated DEGs. In the GL *vs.* GLCK comparison group, 1911 up-regulated and 3963 down-regulated DEGs were identified. To observe the differences between varying leaf colors of the wide-type ‘A18’ and mutant ‘*ytg-2*’, multiple comparisons were performed within the sample set, with a total of 18,338 DEGs in the multiple comparison groups (YL *vs.* YGL *vs.* GL *vs.* YLCK).

**Fig. 6.**
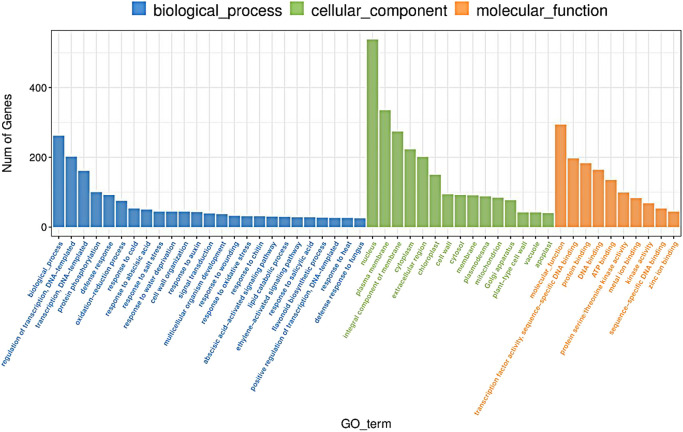
GO enrichment analysis for both the wild-type ‘A18’ and mutant ‘*ytg-2*’. The abscissa represents the GO classification, and the left side of the ordinate represents the number of genes.

To determine the main biological functions exercised by DEG, we performed GO and KEGG enrichment analyses. The DEGs were sequentially compared with GO and KEGG databases to obtain GO terms and KEGG pathways associated with their expression patterns. The top 10 significantly enriched GO were determined for each of the three ontologies: biological process, molecular function, and cellular component. Specifically, 483 DEGs were annotated to the biological process category, 804 DEGs to the molecular function category, and 1842 DEGs to the cellular component category. Notably, the majority of the genes in the biological process category were enriched in the functional group of protein phosphorylation (GO:0006468). In the molecular function category, the most prominent genes were associated with protein serine/threonine kinase (GO:0004674), transcription factor activity, and sequence-specific DNA binding. In the cellular component category, the DEGs were primarily enriched in the functional groups of the extracellular region (GO:0005576) and plasma membrane (GO:0005886). In the multiple comparison groups (YL *vs.* YGL *vs.* GL *vs.* YLCK), differentially expressed genes were mainly enriched in cellular fractions, such as chloroplast envelope, chloroplast thylakoid, chloroplast, and chloroplast stroma. This indicated a close association between the leaf color mutation in ‘*ytg-2*’ and the expression of genes involved in chloroplast development, photosynthesis, and energy metabolism. Fig. 6 demonstrated that the yellow-to-green mutant ‘*ytg-2*’ had more differential genes that regulate chlorophyll synthesis by regulating chloroplast formation and development and other related synthetic pathways. KEGG enrichment analysis revealed that DEGs were mainly enriched in phytohormone signaling pathways (ko04057), amino and nucleotide sugar metabolism (ko00520), phytopathogen interactions (ko04626), phenylpropanoid biosynthesis (ko00940), starch and sucrose metabolic pathways (ko00500), and other energy metabolism- and carbohydrate metabolism-related pathways. In addition, secondary material metabolic pathways including flavonoid biosynthesis (ko00941), anthocyanin biosynthesis (ko00942), zeatin biosynthesis (ko00908), fatty acid elongation (ko00062), fatty acid metabolism (ko01212), linoleic acid metabolism (ko00591), cyanogenic amino acid metabolism (ko00460), and phenylpropane biosynthesis (ko00940) were also enriched among the DEGs. Moreover, the DEGs were also enriched in photosynthesis-related pathways, such as porphyrin and chlorophyll metabolism, photosynthesis-antenna protein, pyruvate metabolism, glycolysis/gluconeogenesis, pentose and glucuronide interconversion-related pathways, as well as glutathione metabolism and peroxisomal-related pathways. Chlorophyll and carotenoids are the main photosynthetic pigments in photosynthesis and play an important role in photosynthetic capture and light energy transfer. Mutations in leaf color have been shown to cause mutations in genes related to chlorophyll and carotenoid synthesis pathways. This can lead to changes in the corresponding chlorophyll and carotenoid contents of plants, eventually causing variations in plant leaf color phenotypes.

### Screening and analysis of leaf yellow-to-green mutant related DEGS

#### Analysis of genes related to chlorophyll biosynthesis pathway

**Fig. 7.**
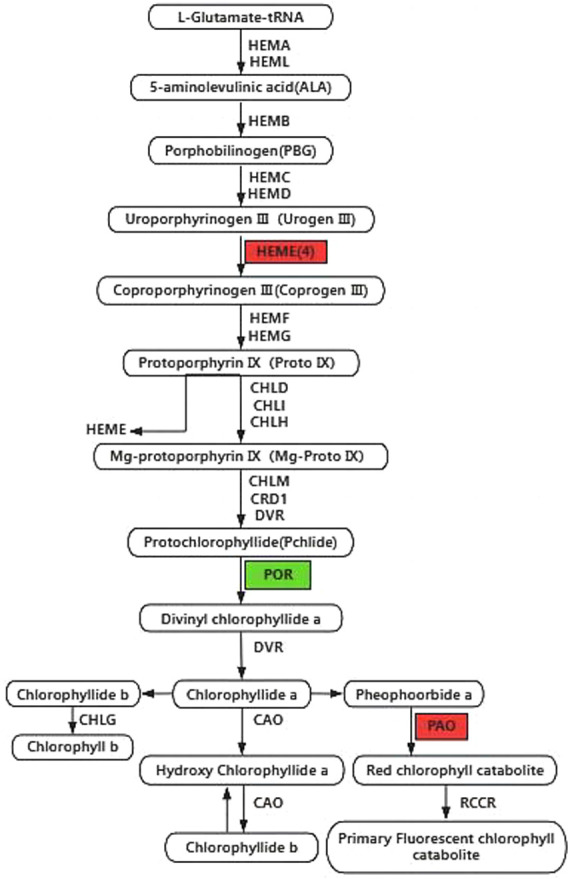
Chlorophyll synthesis pathway. Up-regulated genes are shown in green squares; down-regulated genes are shown in red squares.

**Fig. 8.**
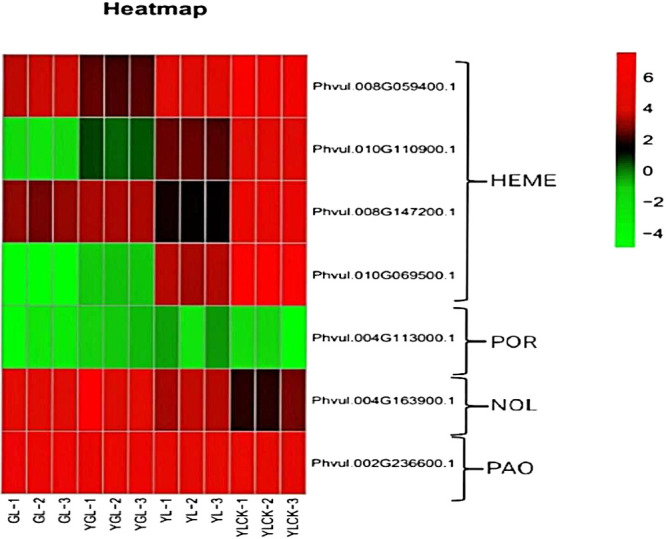
Heat map of gene expression levels within the chlorophyll synthesis pathway.

As shown in Figs. 7 and 8, in the chlorophyll biosynthesis pathway, four genes in the HemE gene family (*Phvul.008G059400*, *Phvul.010G110900*, *Phvul.008G147200*, and *Phvul.010G069500*) were significantly down-regulated in expression and were responsible for the inhibition of Urogen III to Coprogen III synthesis. *POR* (*Phvul.004G113000*) catalyzed the blocked synthesis of protochlorophyllide a to chlorophyllate a, resulting in a reduction in the overall chlorophyll content in the mutant. The *NOL* (*Phvul.004G163900*) gene was significantly up-regulated during the chlorophyll cycle, confirming the accelerated efficiency of the chlorophyll cycle in mutant leaves during the transition to green. This explains the gradual restoration of the chlorophyll a/b ratio to normal levels in the mutant yellow leaves. The up-regulation of a gene associated with *PAO* (*Phvul.002G236600*), a critical enzyme in chlorophyll degradation, suggested a potential connection between the transition from yellow to green and the degradation of chlorophyll content. In summary, the interaction of genes in chlorophyll synthesis and chlorophyll degradation appears to be the primary contributing factor to the decrease in total chlorophyll content. Furthermore, the significant up-regulation of *NOL* gene expression during the chlorophyll cycle led to an accelerated cycle efficiency, resulting in a gradual shift of chlorophyll a and chlorophyll b contents in yellow leaves to normal levels.

#### Analysis of genes related to carotenoid synthesis

**Fig. 9.**
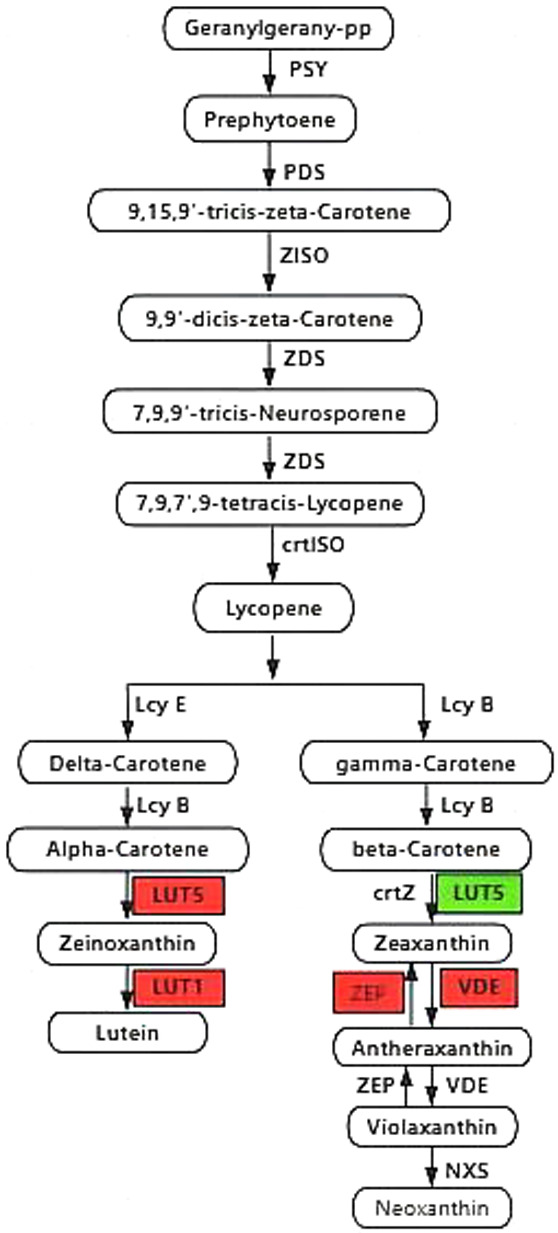
Carotene synthesis pathway. Up-regulated genes are shown in green squares; down-regulated genes are shown in red squares.

**Fig. 10.**
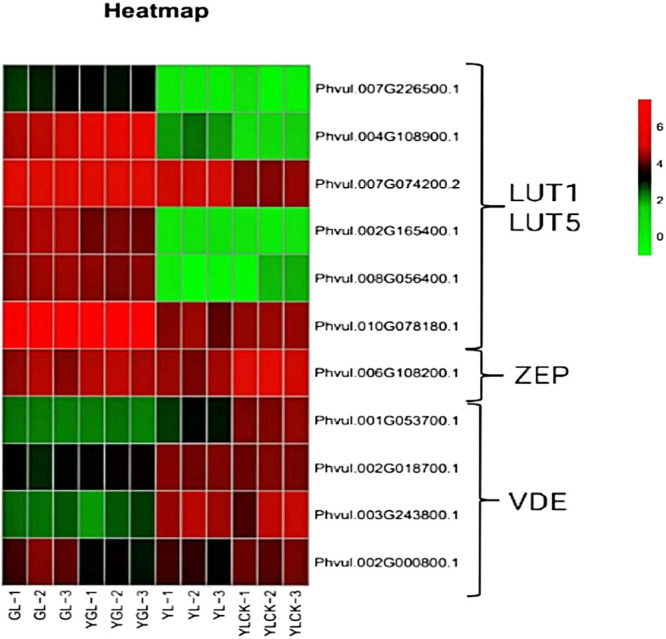
Heat map of gene expression levels within the carotenoid synthesis pathway.

In the carotenoid pathway, the first C40 carotenoid, octa hydro lycopene, is synthesized through the condensation of two *C20 GGPP by PSY* molecules. As shown in Figs. 9 and 10, the lycopene cycle is a key step in carotenoid metabolism, producing different types of carotenoids. The synthetic expression of the two-lycopene cyclase’s lcyE and lcyB genes was significantly down-regulated. This indicated a gradual decrease in carotenoid content during the greening of the mutant leaves. Moreover, subsequent hydroxylation of α-carotene and β-carotene produced lutein (or zeaxanthin) via LUT1, LUT5, CrtR-b, and CrtZ. In this study, there were six DEGs in *LUT1* and *LUT5* (*Phvul.007G226500*, *Phvul.004G108900*, *Phvul.007G074200*, *Phvul.002G165400*, *Phvul.008G056400*, and *Phvul.010G078180*) with a total of two significantly down-regulated genes. The ZEP enzyme plays a crucial role in the lutein cycle, and the gene regulating *ZEP* (*Phvul.006G108200*) was significantly down-regulated in expression. This down-regulation reduced lutein levels. Four genes involved in carotenoid metabolism exhibited significant down-regulattion, including those in *VDE* (*Phvul.001G053700*, *Phvul.002G018700*, *Phvul.003G243800*, and *Phvul.002G000800*). These findings are consistent with the observed decrease in carotenoid content. In conclusion, the lower expression of carotenoid rate-limiting genes, *lcyE* and *lcyB*, in the mutant than in the wild-type likely contributed to their low carotenoid content.

### RT-PCR validation of transcriptome sequencing results

**Fig. 11.**
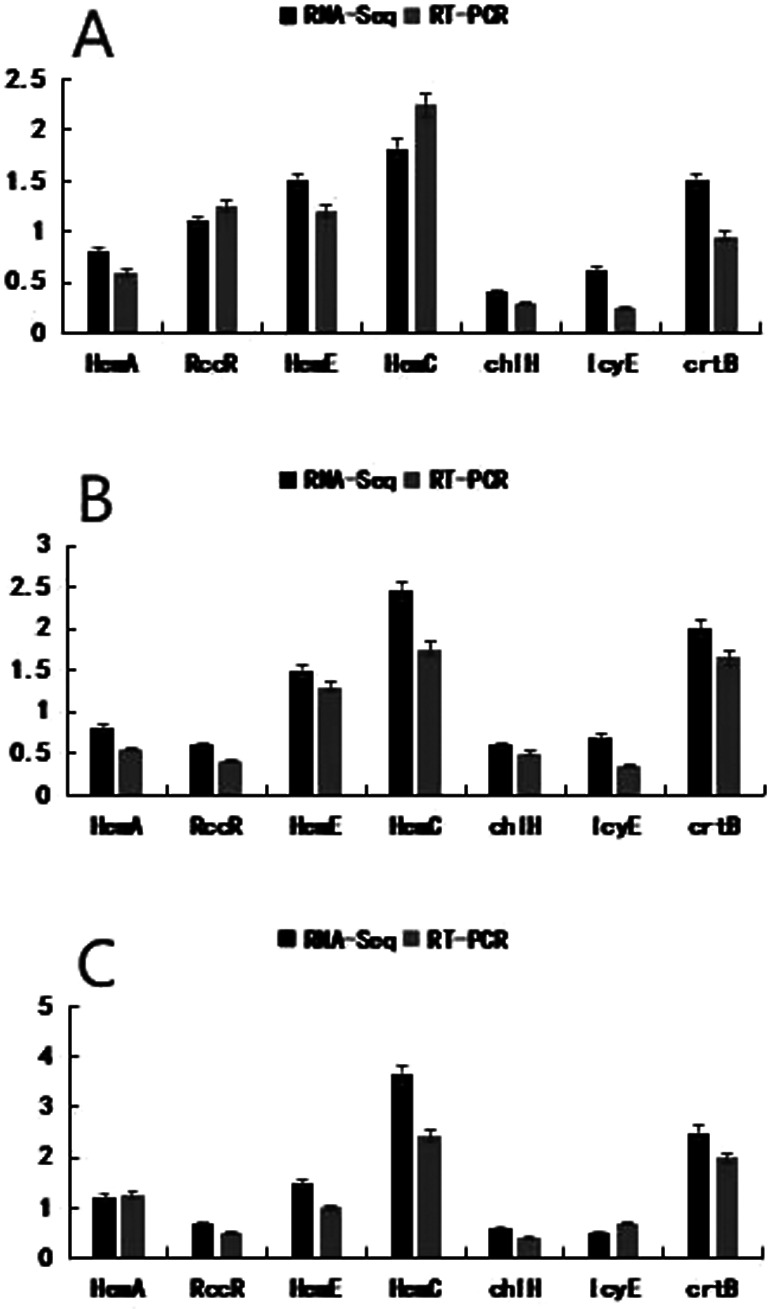
Analysis of qRT-PCR expression levels of genes related to chlorophyll synthesis in three periods of the mutant ‘*ytg-2*’ and wide-type ‘A18’. A) YL *vs.* YLCK; B) YGL *vs.* YGLCK; C) GL *vs.* GLCK.

Based on the transcriptome sequencing results, seven DEGs involved in chlorophyll and carotenoid metabolic pathways were randomly selected for RT-PCR to verify the transcriptome sequencing results. As shown in Fig. 11, the relative expression of all seven genes showed similar trends to the transcriptome sequencing results. This consistency strongly suggests the reliability of transcriptome sequencing data.

## Discussion

Leaf color mutants are valuable materials for investigating chlorophyll synthesis, chloroplast structure and function, and photosynthetic mechanisms. They are also essential for gene mapping, functional genomics, and breeding programs aimed at enhancing the photosynthetic efficiency. In this study, seeds of the ‘A18’ variety were exposed to ^60^Co-γ rays, resulting in the isolation of a stable mutant, ‘*ytg-2*’, after screening for several generations. Observations showed that the leaves initially appeared yellow and gradually turned green as the plants matured. Previous studies have classified such mutants, which exhibit reduced chlorophyll content during early leaf development that normalizes with maturation, as yellow-to-green mutants. Yellowing mutant plants have reduced photosynthetic capacity of leaves and lower carbon assimilation capacity than wild-type plants, which often leads to dwarf plants and weak growth, thereby affecting crop yield. The wheat yellow leaf mutant ‘ygl’ displayed a persistent yellow-green color throughout its development. This mutant showed significantly lower plant height, number of spikes per plant, number of grains per spike, yield per plant, and thousand-grain weight than wild-type plants (Li *et al.* 2013). Similarly, in this study, the mutant ‘*ytg-2*’ exhibited lower plant height, main stem thickness, fresh weight, and leaf area in comparison to the wild-type, resulting in impaired pod development, significantly lower single plant yield, curled and wrinkled pods, and unfilled seeds. Previous studies have highlighted that most leaf yellowing or whitening phenotypes in plants stem from the chloroplast structure or inhibition of chlorophyll synthesis. In this study, it was evident that the total chlorophyll content in the yellow-to-green leaf mutant ‘*ytg-2*’ was significantly reduced, mainly due to the lack of Chl a and Chl b. Further determination of chlorophyll synthesis precursors revealed that the contents of Coprogen III, Proto IX, Mg-Proto IX, and Pchlide were all significantly lower than those of the wild-type ‘A18’, especially the contents of Coprogen III were significantly lower than those of wild-type ‘A18’. This suggested that their synthesis was severely inhibited and could affect the synthesis of subsequent precursors. As the chloroplast thylakoid is the site of photosynthesis, the photosynthetic pigments distributed on it are involved in the absorption, transmission, and conversion of light energy, thus affecting the photosynthetic capacity of plants. Numerous studies have suggested individual or overall decreases in gas exchange parameters, such as Pn, Tr, Gs, and Ci, in the leaf yellowing mutant compared with the wild-type plants. Additionally, certain studies have also indicated that the first three parameters decreased significantly in the mutant, whereas the trend of Ci change was the opposite. The results of this study are aligned with the former. This suggested that the yellow-to-green mutant ‘*ytg-2*’ in the yellow-to-green mutant decreased with the progression of growth.

Changes in plant color are commonly associated with mutations in pigment content, chloroplast development, and photosynthesis. The content and type of pigments provide plants with rich color. Chlorophyll, including Chl a, Chl b, and Car, imparts white, green, yellow, and other colors to the plants. Chlorophyll content usually affects the color change in plant appearance. The relative proportions of chlorophyll and carotenoids primarily determine the leaf color in plants. Chloroplast deficiency directly leads to the production of albino mutants, whereas plants with chlorophyll deficiency but high carotenoid content owing to blocked chlorophyll synthesis mostly exhibit a yellowing leaf phenotype. The plant chlorophyll synthesis pathway comprises a complex regulatory network that can interact with other cellular processes (Li *et al.* 2015, Masuda and Takamiya 2004). Similar to the model plant *Arabidopsis thaliana*, chlorophyll synthesis in most plants is a metabolic pathway from glutamine tRNA to Chl a and Chl b, which consists of 27 genes encoding 15 enzymes (Meier *et al.* 2011). The pathways for chlorophyll synthesis and degradation in mature plants are highly coordinated processes catalyzed by a series of enzymes encoded by nuclear genes. Mutations in any of the enzyme-encoding genes can lead to a decrease in the chlorophyll content and subsequent changes in leaf color. For example, the three genes CHLH, CHLD, and CHLI constitute magnesium chelatase, and mutations in CHLH have been reported to cause yellow or yellow-green mutants in Arabidopsis and rice (Jung *et al.* 2003, Mochizuki *et al.* 2008). In addition, chlorophyll-deficient mutants associated with the CHLI gene have been identified in soybean (Li *et al.* 2016a). In rice and tobacco leaf color mutants, the diminished expression of *POR* genes during chlorophyll synthesis significantly reduced chlorophyllase synthesis (Masuda *et al.* 2002, Sakuraba *et al.* 2013). In the this study, the *POR* (*Phvul.004G113000*) genes encoding porphyrins and chlorophyll were significantly down-regulated in the yellow leaf mutants, which could contribute to the decreased content of Chl a and Chl b. The significant up-regulation of the *NOL* (*Phvul.004G163900*) gene during the yellow-to-green transition confirmed that the chlorophyll cycle efficiency was accelerated during the leaf transition to green, explaining the gradual restoration of the Chl a/b ratio to normal levels in the mutant yellow leaves.

The lack of chlorophyll has been reported in various leaf color mutations, including those in Arabidopsis, Brassica, and sunflower (Azarin *et al.* 2020, Li *et al.* 2017, 2018, Maekawa *et al.* 2015). In this study, the chlorophyll a and total chlorophyll b contents in yellow leaves were significantly lower than those in green leaves. In higher plants, chlorophyll synthesis proceeds according to the following pathway: Glu → ALA → PBG → Urogen III → Proto IX → Mg-proto IX → Pchlide → Chl a → Chl b (von Wettstein *et al.* 1995). Any disruption in the enzymatic steps of this pathway can result in a reduction in the corresponding synthesized substances and a decrease in the content of Chl a and Chl b. This can lead to a change in plant leaf color. For instance, in the ‘pylm’ mutant of cabbage, chlorophyll biosynthesis is blocked during chloride esterification (Zhang *et al.* 2016, 2020). It has been suggested that chlorophyll deficiency is caused by blocked synthesis of prodegenerate chlorophyll to degenerate chlorophyll. In this study, we discovered that the total chlorophyll contents in the mutant ‘*ytg-2*’ were significantly lower than those in the wild-type ‘A18’ in measuring the changes of chloroplast synthesis precursors. Chlorophyll biosynthesis in the mutant ‘*ytg-2*’ appears to be inhibited during Urogen III to Coprogen III synthesis, which can lead to a suppression of chlorophyll biosynthesis metabolism and a subsequent reduction in chlorophyll content within mutant ‘*ytg-2*’ leaves. Additionally, the expression of chlorophyll b reductase (*NOL*), which is associated with chlorophyll degradation, was up-regulated in mutant leaves. Sequencing results revealed that chlorophyll biosynthesis was catalyzed by 15 enzymes based on comparisons between ‘A18’ and ‘*ytg-2*’ in yellow and green leaves. Any disruption in this process leads to chlorophyll deficiency and the development of leaf color mutations. In summary, genes involved in chlorophyll biosynthesis and chloroplast biogenesis were repressed in young yellow leaves. As the leaves mature, these genes become active in certain regions, resulting in light green leaves. However, the molecular mechanisms underlying this process require further investigation.

Etiolation or leaf yellowing typically results from reduced photosynthetic efficiency and inhibition of photosynthetic product synthesis, which can hinder plant growth. However, recent advances in the study of leaf color mutants have expanded their applications in both theoretical research and practical breeding. The yellow-to-green mutant is a readily identifiable phenotypic trait and serves as a marker for crossbreeding, which improves breeding efficiency and offers significant potential in crop genetics and breeding. For example, the yellow-green leaf rice mutant has been used to select male sterile lines for Japonica three-line breeding. Variations in leaf color often involve fundamental areas of plant research, such as chloroplast development, light-induced morphogenesis, and chlorophyll metabolic pathways. Utilizing leaf color variation mutants as research models facilitates the study of gene function, and transcriptome analysis can uncover gene interactions.

## Author Contribution Statement

D.L., X.Y., and R.J. conceived, designed, and performed the experiments; Z.W. wrote the manuscript; G.F. contributed to the experimental materials and field planting; and Z.Y. and C.L. revised the manuscript and performed data analysis. All authors have read and agreed to the published version of the manuscript.

## Supplementary Material

Supplemental Table

Supplemental Text

## Figures and Tables

**Table 1.** qRT-PCR primer sequences

Gene Name	Upstream Primer	Downstream Primer
Phvul.008G185100	TTGGACAGGGAGTGAATGGC	CACCGAATGGCCCCTCTATG
Phvul.008G280200	GATTTGCCTCCCCGGAAAGA	TCCAGAGCCTGCCTATGAGT
Phvul.001G248800	TTCACACAGATGCCCAGGTC	GGTGCCCCAACAAAACCAAG
Phvul.002G034500	TCCGTCTTCCAAATGCCACA	CTTGTTTGGACATTGCCCCG
Phvul.001G132200	TTGCTGCACTTGATCGTCCT	TTCCACCGTTCTCAGCCTTC
Phvul.009G230300	TGAGGTTGCGGAGGAAGAAC	GACTCGACACACCTGCGTAA
Phvul.006G024100	ATTGATGACCCAAGAGCGCA	TCTGATAGCCCAGCTTGTGC
Actin	GAAGTTCTCTTCCAACCATCC	TTTCCTTGCTCATTCTGTCCG

**Table 2.** Comparative analysis of yield-related traits between wild-type ‘A18’ and mutant ‘*ytg-2*’

	Pod length (cm)	Pod width (cm)	Single plant yield (cm)	Number of pods per plant (pods)	Number of seeds per pod
A18	14.72 ± 0.79	1.16 ± 0.77	112.3 ± 15.33	62.50 ± 9.39	5.50 ± 0.55
*ytg-2*	10.47 ± 1.95**	1.09 ± 0.12	60.81 ± 5.52**	30.83 ± 4.54**	2.17 ± 0.89**

**Table 3.** Number of reads based on Illumina sequencing data in 18 libraries of mutant *ytg-2* and wide-type ‘A18’

Sample	Raw Data	Valid Date	Valid Ratio	Q20%	Q30%	GC content%
GL_1	48712168	47531016	97.58	99.91	97.81	44.5
GL_2	51435664	49431182	96.1	99.91	97.7	44.5
GL_3	51935578	50606234	97.44	99.91	97.77	44.5
GLCK_1	43133654	42311430	98.09	99.91	97.82	43.5
GLCK_2	45812232	44907750	98.03	99.91	97.89	43.5
GLCK_3	45321560	44445676	98.07	99.91	97.81	43.5
YGL_1	54175566	53033612	97.89	99.92	97.8	44.5
YGL_2	54007848	52753162	97.68	99.91	97.79	44.5
YGL_3	49654402	48707388	98.09	99.92	97.72	44.5
YGLCK_1	54284518	52839470	97.34	99.91	97.89	43.5
YGLCK_2	54655244	53386658	97.68	99.9	97.85	43.5
YGLCK_3	46763122	45771434	97.88	99.9	97.87	43.5
YL_1	41852642	41135690	98.29	99.91	97.89	44
YL_2	36734886	36100268	98.27	99.93	97.98	44.5
YL_3	52098956	50727238	97.37	99.92	97.83	44.5
YLCK_1	49093930	48058414	97.89	99.91	97.7	43.5
YLCK_2	41055912	40236372	98	99.91	97.8	43.5
YLCK_3	34110222	33453190	98.07	99.92	97.84	43.5
