# Supplementary material for: Leaf color variation mechanism of the yellow-to-green mutant ‘ytg-2’ in Phaseolus vulgaris L
Source: Breed Sci. 2025 Jun 11;75(3):155–67. doi: 10.1270/jsbbs.24018 (PMC12457786; doi:10.1270/jsbbs.24018)
Supplement: Supplementary file 1 — Supplemental Table [file 75_155_s1.pdf]

doi: 10.1270/jsbbs.24018

Supplemental **Table 1**

Name	Abbreviation
**Genes related to chlorophyll synthesis**	
glutamyl-tRNA synthetase	HemX
glutamyl-tRNA reductase	HemA
glutamate-1-semialdehyde 2, 1-aminomutase	HemL
porphobilinogen synthase	HemB
hydroxymethylbilane synthase	HemC
uroporphyrinogen-IⅡ synthase	HemD
uroporphyrinogen decarboxylase	HemE
coproporphyrinogen IⅡ oxidase	HemF
protoporphyrin/coproporphyrin ferrochelatase	HemH
oxygen-independent coproporphyrinogen IⅡ oxidase	HemN
protoporphyrinogen/coproporphyrinogen IⅡ oxidase	HemY
magnesium chelatase subunit H	chlH
magnesium-protoporphyrin O-methyltransferase	chIM
magncsium-protoporphyrin IX monomethyl ester cyclase	chIE
Protochlorophyllide reductase	POR
divinyl chlorophyllide a 8-vinyl-reductase	DVR
chlorophyllide a oxygenase	CAO
chlorophyllide b reductase	NOL
magnesium chclatase subunit H	CHLH
chlorophyll/bacteriochlorophyll a synthase	chlG
7-hydroxymethyl chlorophyll a reductase	HCAR
pheophorbide a oxygenase	PAO
red chlorophyll catabolite reductase	RCCR
**Carotenoid biosynthesis pathway**	
15-cis-phytoene synthase	PSY
15-cis-phytoene desaturase	PDS
zeta-carotene isomerase	Z-ISO
zeta-carotene desaturase	ZDS
prolycopene isomerase	crtISO
lycopene epsilon-cyclase	lcyE
lycopene beta-cyclase	lcyB
beta-ring hydroxylase	LUT
beta-carotene 3-hydroxylase	crtZ
zeaxanthin epoxidase	ZEP
violaxanthin de-epoxidase	VDE
zinc D-Ala-D-Ala dipeptidase	NXS
**Chlorophyll synthesis precursor**	
5-Aminolevulinate	ALA
Porphobilinogen	PBG
Uroporphyrinogenlll	Urogen III
Coproporphyrinogen III	Coprogen III
Protoporphyrinogen IX	Proto IX
Mg-ProtoporphyrinIX	Mg-Proto IX

Protochlorophyllide	Pchlide
**other**	
Proline	Pro
Gene Ontology	GO
Kyoto Encyclo pedia of Genes and Genomes	KEGG
Differential Expressed Genes	DEGs
stomatal conductance	Gs
IntercellularCO2concentration	C1
transpiration rate	E
net photosynthesis rate	Pn
Chlorophyll a	chl a
Chlorophyll b	chl b
Chlorophyll	chl
Carotenoid	car
nuclear-encoded photosynthetic gene	Lhcb
inhibitor lincomycin	LIN
ABC transporter protein I family member 1	ABCI1
formyl glycine rib peptide aminotransferase	FGAMS
Fragments Per Kilobase per Million	FPKM
