# Supplementary material for: Leaf color variation mechanism of the yellow-to-green mutant ‘ytg-2’ in Phaseolus vulgaris L
Source: Breed Sci. 2025 Jun 11;75(3):155–67. doi: 10.1270/jsbbs.24018 (PMC12457786; doi:10.1270/jsbbs.24018)
Supplement: Supplementary file 2 — Supplemental Text [file 75_155_s2.pdf]

doi: 10.1270/jsbbs.24018

Supplementary Text 1 RNA extraction operation steps (Takara Biotechnology, Dalian, China)

1. Homogenization treatment
Plant leaves (50–100 mg) were quickly ground into a fine powder using liquid nitrogen. Then, 450 μL RL was added (ensuring that β-mercaptoethanol was included before use), then vortexed vigorously to mix.

2. The mixture was transferred onto the filter column CS, placed in a collection tube, centrifuged at 12000 rpm (~13400×g) for 2–5 min. The supernatant was carefully transferred into an RNase-free centrifuge tube, avoiding contact with the cell debris sediment.

3. Anhydrous ethanol, at 0.5 times the volume of the supernatant (usually 225 μL), was slowly added and mixed thoroughly. If precipitation occurred, both the solution and precipitate were transferred into adsorption column CR3. The mixture was centrifuged at 12000 rpm (~13400×g) for 30-60 seconds, the waste liquid discarded, and adsorption column CR3 was placed into the recovery header.

4. Then, 350 μL of protein removal solution RW1 was added to adsorption column CR3. It was centrifuged at 12000 rpm (~13400×g) for 30–60 seco, the waste liquid discarded, and adsorption column CR3 was returned to the recovery header.

5. The DNase I working solution was prepared by mixing 10 μL of DNase I storage solution with 70 μL of RDD buffer in a new RNase-free centrifuge tube.

6. Then, 80 μL of the DNase I working solution was added to the center of adsorption column CR3 and left to incubate at room temperature for 15 min.

7. Protein removal solution RW1 (350 μl) was added to adsorption column CR3, which was then centrifuged at 12000 rpm (~13400×g) for 30–60 s. The waste in the collection tube was discarded, and the adsorption column CR3 was placed in the recovery header.

8. Rinsing solution RW (500 μL) was added to adsorption column CR3 after confirming ethanol was present. It was left at room temperature for 2 min, centrifuged at 12000 rpm (~13400×g) for 30-60 s, the waste liquid was discarded, and the adsorption column CR3 was placed in the recovery header.

9. Step 8 was repeated.

10. The column was centrifuged at 12000 rpm (~13400×g) for 2 min, and the waste liquid was discarded. The adsorption column CR3 was allowed to air dry at room temperature for several minutes to remove any residual rinsing solution in the adsorption material.

11. The adsorption column CR3 was transferred to a new RNase-free centrifuge tube. RNase-free ddH_2O (30–100 μl) was added to the center of the adsorption membrane, allowed to stand at room temperature for 2 min, then centrifuged at 12000 rpm (~13400×g) for 2 min to obtain the RNA solution.
